# Neurophotonics Approaches for the Study of Pattern Separation

**DOI:** 10.3389/fncir.2020.00026

**Published:** 2020-06-09

**Authors:** Cristian Morales, Juan Facundo Morici, Magdalena Miranda, Francisco Tomás Gallo, Pedro Bekinschtein, Noelia V. Weisstaub

**Affiliations:** ^1^Departamento de Psiquiatria, Centro Interdisciplinario de Neurociencia, Pontificia Universidad Católica de Chile, Santiago, Chile; ^2^Instituto de Neurociencias Cognitiva y Traslacional (INCYT), Concejo Nacional de Investigaciones Científicas y Tecnológicas (CONICET), Instituto de Neurología Cognitiva (INECO), Universidad Favaloro, Buenos Aires, Argentina

**Keywords:** memory, pattern separation, optogenetics, calcium imagaing, granule cells, mossy cells, interneuron, adult born granule cells

## Abstract

Successful memory involves not only remembering over time but also keeping memories distinct. Computational models suggest that pattern separation appears as a highly efficient process to discriminate between overlapping memories. Furthermore, lesion studies have shown that the dentate gyrus (DG) participates in pattern separation. However, these manipulations did not allow identifying the neuronal mechanism underlying pattern separation. The development of different neurophotonics techniques, together with other genetic tools, has been useful for the study of the microcircuit involved in this process. It has been shown that less-overlapped information would generate distinct neuronal representations within the granule cells (GCs). However, because glutamatergic or GABAergic cells in the DG are not functionally or structurally homogeneous, identifying the specific role of the different subpopulations remains elusive. Then, understanding pattern separation requires the ability to manipulate a temporal and spatially specific subset of cells in the DG and ideally to analyze DG cells activity in individuals performing a pattern separation dependent behavioral task. Thus, neurophotonics and calcium imaging techniques in conjunction with activity-dependent promoters and high-resolution microscopy appear as important tools for this endeavor. In this work, we review how different neurophotonics techniques have been implemented in the elucidation of a neuronal network that supports pattern separation alone or in combination with traditional techniques. We discuss the limitation of these techniques and how other neurophotonic techniques could be used to complement the advances presented up to this date.

## Introduction

Research in the memory field has been interested not only in the ability to remember over time but also in the capacity to keep memories differentiated and resistant to confusion. To evoke a memory, our brain needs to integrate the information it receives from the environment. This integration is important for coding the general structure of the environment and abstracting it from the specificities of individual events, which allows us to generalize to novel situations. This ability to separate memory components into unique representations was postulated to rely on a computational process known as “pattern separation” (McClelland et al., 1995; Norman and O’Reilly, 2003). Computational models define this process as a transformation of the correlated input information into an orthogonal output (Marr, 1971; Treves and Rolls, 1994; Ranganath, 2010). According to these theories, the correct storage and retrieval of memories require the stored of the information in nonoverlapping representations. Because episodic memory implies learning about unique events and avoid interference, being able to differentiate them is particularly important for this kind of memories so that storing new information does not lead to overwriting previously stored ones. For this reason, pattern separation is proposed as an essential component for the storage of differentiated representations of episodic memories and as such has been mainly studied in the hippocampus (HP; Ranganath, 2010).

**Figure 1:**
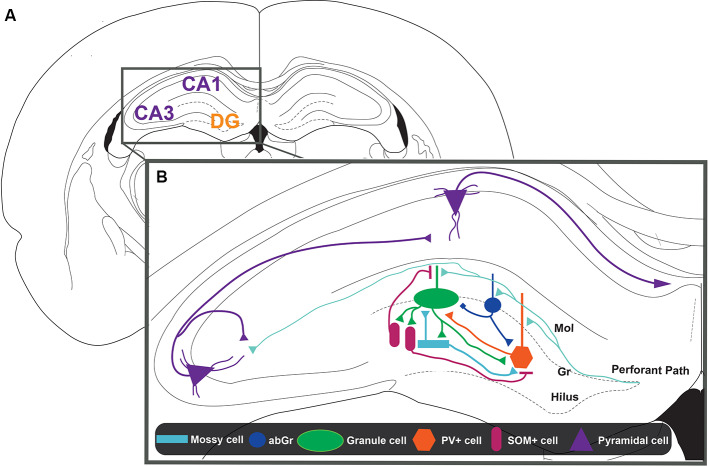
Representation of the dentate gyrus (DG)-CA3 circuit.** (A)** Schematic representation of a coronal slice from rat brain. The hippocampus (HIP) is constituted by cornu ammonis regions 1 and 3 (CA1 and CA3, in violet) and the DG region (DG, in orange). **(B)** Zoom inset from the HIP. The main inputs to the HIP come from the layers 2/3 of the entorhinal cortex (EC) that constitute the perforant path (PP, light blue lines). The information coming from the EC could project to the CA3 pyramidal layer directly or indirectly by making and intermediate synapse on the granule cells (GCs, green ellipse) located in the granular layer (Gr) of the DG. Mossy cells (MCs, ligth blue rectangle) and adult-born GCs (abGC, blue circle) would be the first neurons activated and could initiate pattern separation. The abGCs can modulate the activation of GCs through a direct connection, which is excitatory or inhibitory, depending on the activity pattern of entorhinal input. On the other hand, abGCs and MCs can inhibit GCs by recruitment of parvalbumin containing interneurons (PV+, orange hexagon). Also, the activity of PV+ interneurons is modulated by somatostatin containing interneurons (SOM+, pink ellipse) and by GCs itself. SOM+ interneurons can directly inhibit GCs, specifically distal dendrites, where contextual information arrives. Thus, the interaction between the different cell types present in the DG defines the activity of GCs, which project to CA3. Finally, the CA3 pyramidal cells project through the Schaffer collaterals to the CA1 pyramidal cells forming the main hippocampal output into layer 5 of the EC.

The HP is one of the structures that constitute the medial temporal lobe, and it has been associated with the pattern separation process. Classically, four regions have been identified in the HP that have distinct anatomical, physiological, and genetic characteristics (Figure 1): the regions cornu ammonis 1, 2, and 3 (CA1, CA2, and CA3) and the dentate gyrus (DG). Computational models first suggested the potential importance of the DG for this cognitive function. The attractor system present in CA3 would be favored by a previous decorrelating process in the DG that could increase the storage capacity of the CA3 system (Amaral et al., 1990; Rolls et al., 1998). The presence of a highly inhibited DG structure or subregion, with a five-time greater number of cells than the upstream entorhinal cortex (EC), and divergent connectivity toward the CA3 region appears as the perfect structure to be able to achieve this randomizing function (Amaral et al., 1990; Jung and McNaughton, 1993; Chawla et al., 2005; Leutgeb et al., 2007). The potential adaptive role of this putative function was immediately appreciated because very similar events could lead to different outcomes and being able to judge this is crucial for our cognitive versatility.

Many tasks have been developed to show the relevance of the pattern separation process for cognition (Gilbert et al., 1998, 2001; Clelland et al., 2009; Toner et al., 2009; Creer et al., 2010; Bekinschtein et al., 2013). Gilbert et al. (2001) found that the DG ablation leads to a deficit in the discrimination of two similar positions based on distal cues. This deficit was not observed if the separation between the positions was greater. These results were confirmed in subsequent studies (Goodrich-Hunsaker et al., 2005), strongly supporting the role of the DG in pattern separation. The gradual observed impairment indicates a failure in pattern separation at the behavioral level. Consistently, McHugh et al. (2007) found, using a genetic approach, that mice lacking the essential NR1 subunit of the N-methyl-D-aspartate receptor (NMDA receptors; rNMDA) in GCs of the DG could not distinguish two similar contexts during a fear-conditioning task, although their performance in a regular task of contextual fear conditioning was normal (McHugh et al., 2007). Thus, the results indicate that DG participates in the discrimination of spatial or contextual information. The experiments commented above allowed to postulate the existence of a pattern separation process, which can be deduced from the behavioral performance (e.g., good execution on the pattern separation task) have correctly occurred and can only hypothesize about the existence of an underlying circuit-level process that supports this kind of cognitive discrimination.

Human studies indicate that patter separation takes place in the DG. In studies using high-resolution functional magnetic resonance imaging to measure brain activity during incidental memory encoding (Bakker et al., 2008; Lacy et al., 2011), authors found that CA3/DG activity was highly sensitive to small changes in the input. In such studies, the interpretation is that DG amplifies the differences between highly similar objects, thus generating highly dissimilar and nonoverlapping representations. Then, the evidence accumulated from animal and human studies supports the theoretical models proposed for the DG to be involved in pattern separation. As from the mechanism underlying this process, theoretical models proposed that the correct occurrence of the pattern separation process requires low excitability of GCs to induce the orthogonalization of memory representations (Treves and Rolls, 1992; Rolls and Kesner, 2006; Rolls, 2013) The low excitability would permit a small number of GCs to represent an episode and then decreasing the possibility of superposition between similar representations (Rolls, 2013). Also, orthogonalization could be a mechanism that forces distinct GCs to be active in the codification of similar episodes (Rolls, 2013). Lesion studies by electrolytic or histochemical techniques (Gilbert et al., 1998, 2001; Goodrich-Hunsaker et al., 2005; Hunsaker et al., 2008) and traditional electrophysiological techniques (Leutgeb et al., 2007; Neunuebel and Knierim, 2014) support this theoretical model. However, the exact mechanism by which pattern separation occurs remains unresolved mainly for technical limitations. Over the last decades, the implementation of new technologies in the study of biologically relevant questions opens a new window of opportunity to tackle in undertaking the complexity of the DG circuitry. Particularly, photonic techniques are one of the most used in neuroscience research (Torricelli et al., 2014). Their popularity comes from their characteristics, such as their adaptability to different settings and their versatility to study different problems, from the cellular to the behavioral level, as well as their high temporal and structural accuracy for cell-specific activity measurement and activity intervention (Cho et al., 2016). In this scenario, the usage of neurophotonics has shown some advantages over previous techniques in the study of the DG microcircuit involved in pattern separation. Regarding the complexity of this issue due to the cell-type variability proposed to be involved during the pattern separation process, it is important to try and contrast the theoretical models with the empirical evidence. In this review article, we focus primordially on the evidence obtained by neurophotonics techniques.

## Implications of Memory Engram Theory in Pattern Separation Process

Pattern separation computational models (Rolls and Kesner, 2006; Rolls, 2013) propose that orthogonalization implicates the activation of different GCs within the total population in different contextual experiences. Empirical experiments have supported this statement. Using the expression of an activity-dependent gene, like the activity-regulated cytoskeleton-associated protein (ARC), Chawla et al. (2005) found that when mice were exposed to two different environments, ARC was expressed in two different sets of neurons. Similar results were obtained for ZIF268 in an experiment where mice were allowed to explore the same environments but with two different motivations (Satvat et al., 2011). Both results indicate that in the DG an orthogonalization process occurs not only to encode different contextual features but also to encode differences between the experiences *per se*. This activation of different cell populations appears to be specific to situations that require differentiation because when the mice explored the same environment with the same motivation, the sets of zif268-positive cells obtained were significantly overlapped (Satvat et al., 2011). This suggests a correspondence between the behavioral experience and the subset of cells that encode it. Interestingly, the representational differentiation also occurs in the CA3 region but not in the CA1 region (Leutgeb et al., 2005, 2007) of the HP, This differential recruitment highlights the differentiation process as a DG and/or CA3 region property.

The existence of the neural substrate of memories that make us unique and unrepeatable individuals has been a matter of discussion for over a century. Richard Semon proposed the existence of physical changes in the brain generated by the encoding of new information and called them “engrams” (Tonegawa et al., 2015). Engrams are commonly defined as a set of cells that are synchronously activated during the encoding of a particular experience resulting in the storage of this new information (Wang, 2019). Semon’s idea was too progressive for his time to experimentally contrast it, although it has changed in the last decades, when several publications supporting the engrams theory have appeared (for a review see Bocchio et al., 2017; Tanaka and McHugh, 2018 and Josselyn and Tonegawa, 2020). As predicted by Martin and Morris (2002): “In our view, the final test of any hypothesis concerning memory encoding and storage must be a mimicry experiment, in which apparent memory is generated artificially without the usual requirement for sensory experience, or indeed any form of experience, during learning. […] In another sense, such an experiment would constitute a critical test that changes in synaptic efficacy are sufficient for memory, rather than merely necessary.” Neurophotonics give us, for the first time, the opportunity to test the engram hypothesis. In the following section, we will try to address some of the advances made in the engram research area by focusing on the use of neurophotonics techniques.

### Neurophotonics in the Development of Engrams Theory

It was not until a few years ago that huge steps were taken in the quest to identify memory engrams, thanks to the generation of different behavioral, molecular, genetic, and optic tools (Josselyn and Tonegawa, 2020). Particularly, optogenetics was the first neurophotonic technique used to tackle this question. Liu et al. (2012) injected an adenoassociated virus (AAV) AAV9-TRE-ChR2-EYFP in the DG of a c-fos–tTA mice. The main idea of this strategy was to express channelrhodopsin 2 (ChR2, excitatory opsin) in the DG cells that were active during the training phase of a fear-conditioning task (Liu et al., 2012; c-fos will be recruited and drive the expression of tTA.) They showed that activating this population of DG cells was enough to trigger the reactivation of the fear response. These results indicated that reactivating the cells recruited during the encoding of a fear memory was sufficient to retrieve and express this memory. This finding was supported and expanded when Ramirez et al. (2013) were able to create a false memory. By reactivating the DG cells recruited during the encoding of a neutral context (context A) while the animal received a shock at a different one (context B), the authors were able to create a false association between context A and the delivery of the electrical shock (Ramirez et al., 2013). Interestingly, this phenomenon was observed in other parts of the brain, such as the olfactory bulb (Vetere et al., 2019) and the amygdala (Redondo et al., 2014), suggesting an underlying common neural mechanism. To study the stability of the contextual component of a fear memory within the neuronal representation in the HP, Ghandour et al. (2019) performed *in vivo* Ca^2+^ imaging of putative engram and nonengram cells in the CA1 region of the HP at posttraining sessions. To do this, they injected a TRE-KikGR lentivirus into the CA1 (to label engram cells) of a Thy1-G-CaMP7 × c-fos–tTA double transgenic mice. KikGR is a highly effective fluorescent protein that could photoconvert from green to red upon the exposure to 365-nm light without affecting the Ca^2+^ imaging signal. Its expression was controlled in an activity and time-dependent manner (by the c-fos–tTA construct). G-Camp7 is a very sensitive calcium sensor. Then, by combining both tools, they could identify the activated engram cells during the recordings. They observed that the total activity pattern of the engram cells during learning was more stable across postlearning memory processing than the activity of the nonengram cells. However, as far as we know, this kind of experiments was not yet performed in the DG. Nevertheless, these results suggest that neurophotonics is a powerful instrument in the quest and identification of memory engrams and their role in the different memory stages of normal and pathological conditions (Roy et al., 2016; Denny et al., 2017).

The capacity of the brain to maintain differentiated engrams could be really useful for the storage of overlapping memories (Deng et al., 2013). Then, understanding how a particular subset of cells is recruited to be part of an engram is indeed important. It has been shown that neuron excitability could be enhanced by CREB expression (Han et al., 2009; Rao-Ruiz et al., 2019), helping with the engram allocation into that particular subset of neurons (Zhou et al., 2009; Kim et al., 2013; Yiu et al., 2014). With this background in mind, Rashid et al. (2016) asked if two fear-conditioning episodes closely in time could recruit similar neuronal populations in the lateral amygdala. They observed that the overlapping between *arc* and *homer1*, two immediate early genes, mRNA increased. Moreover, there was interest in dissecting if the excitability of a particular neuronal ensemble was sufficient to direct the memory allocation to those neurons. To do this, they guide the expression of halorhodopsin (NpHR3.0, inhibitory opsin) and ChR2 to inhibit or excite the activity of the same neuronal population before the first fear conditioning. The main objective behind this experimental design was to bidirectionally modulate the excitability of the transfected neurons to enhance or decrease the degree of overlapping between memory engrams. They observed that the optogenetics manipulations had effects over the engram overlapping outcomes only when the two fear-conditioning episodes were generated within a limited time frame. This result suggests that, depending on the temporal proximity between two slightly different experiences, the neuronal ensemble recruited by both of them could be similar. This experiment suggests that, at least in the amygdala, the time interval between two similar experiences is crucial in the ability to generate distinct memory engrams and implies that this structure might not have the computational ability to use pattern separation as a disambiguating process. This kind of experimental setting would be really useful to dissect if pattern separation happening in the DG requires the allocation of two similar experiences into different neuronal ensembles. However, to tackle this idea, future experiments should parameterize the similarity of the contexts used during training.

More recently, it has been shown that the enhancement of the engram cells excitability after reactivation is mediated by the internalization of Kir2.1 inward-rectifier potassium channels and the activation of rNMDAs (Pignatelli et al., 2019). It was shown that K2+ inward rectifier currents are negatively modulated by the activation of α-amino-3-hydroxy-5-methyl-4-isoxazolepropionic acid receptor (AMPA receptors) (Houzen et al., 1998; Jones et al., 2000; Schröder et al., 2002). Using optogenetics to tag DG engram cells associated with a fear memory, Ryan et al. (2015) have shown that engram cells present an enhancement of the spine density and rAMPA/rNMDA ratios compared with nonengram cells. Interestingly, this phenotype was depleted when the animals received a protein synthesis inhibitor, anisomycin, classically used to impair memory consolidation. To study more in detail this aspect, a technique called dual-eGRASP has been developed (Kim et al., 2012). The conventional GRASP technique requires two complementary mutant GFP fragments, which are expressed separately on presynaptic and postsynaptic membranes. When the complementary GFP fragments interact within each other at the synaptic cleft, a functional GFP appears. Then, the GFP signal indicates a formed synapse between presynaptic and postsynaptic neurons. Using this approach, it was shown that the CA3–CA1 spine density is enhanced after the training phase (Choi et al., 2018), suggesting that the excitability changes observed happen in the entire engram cell ensemble. The increase of spine density observed in the engram cells was related to the enhancement of the rAMPA/rNMDA ratio and subsequently with the Kir2.1 inward-rectifier potassium channel internalization. Then, these results suggested that changes in the excitability of neurons (Park et al., 2016) that are part of an engram could be key in the mechanism of pattern separation of overlapping memories.

It has been proposed that engram cells from distinctive ensembles spread all over the brain (Kastellakis and Poirazi, 2019). This particularity is associated with the capacity of integrating different features of the encoding experience (Guan et al., 2016). According to this idea, activation of DG engram cells, as a hub in the pattern separation mechanism, could trigger the expression of aversive or appetitive responses that are commonly located in different downstream structures (Redondo et al., 2014; Ramirez et al., 2015; Roy et al., 2017; Chen et al., 2019). Ramirez et al. (2015) showed that chronic optogenetic reactivation of rewarded experiences reverts the depressive behavior in a mouse model. Moreover, the chronic reactivation of the dorsal DG engram cells associated with an aversive experience generates extinction response, whereas the reactivation of the ventral DG engram cells generates an enhancement of the fear response (Chen et al., 2019). However, how memory function emerges from the coordinated activity between all the engram nodes remains a mystery. It has been shown that distinct neuronal populations of the basolateral amygdala participate in giving positive or negative valence (Redondo et al., 2014; Gore et al., 2015) to a particular experience. Then, it was proposed that the reactivation of contextual engram cells located in the DG could guide the reactivation of the valence engram cells associated with guiding the behavioral outcome of a certain experience (Tonegawa et al., 2015). These results are in line with the postulation that reactivation of one of the nodes guides the reactivation of the entire engram. Understanding the mechanisms underlying this feature could be useful for the treatment of different anxiety and mood disorders.

Then, the accumulated evidence proposes that the information in the brain could be stored at specific cell ensembles. In this sense, the differentiation of similar information by generating nonoverlapping engrams is proposed as the material outcome of a pattern separation process. Most of the studies focus their attention on the interaction between excitatory neurons at the time of characterizing the intrinsic properties of engram cells. However, the GCs are not the only glutamatergic cells within the DG and neither the only population within the structure. Then, it is plausible that other cell populations might also be engram cells or at least play important modulatory roles to the main cells. In this regard, inhibitory engrams have been proposed to be important for specific memory reactivation (for a review, Barron et al., 2017). In the following sections, we discuss the role of different cell types in the DG circuit involved in pattern separation.

## Dentate Gyrus Glutamatergic Cells Participation in Pattern Separation

### Usage of Neurophotonics in the Study of the Spatial Codification of Granule and Mossy Cells

One of the most studied hippocampal function is its involvement in spatial memory encoding. Lesions or pharmacological interventions on the DG impair the performance in different spatial memory tasks (Gilbert et al., 1998, 2001; Goodrich-Hunsaker et al., 2005, 2008), suggesting a role of this structure during the storage or recall of this kind of memory. Moreover, electrophysiological recordings have shown the existence of place cells within the DG (Jung and McNaughton, 1993; Leutgeb et al., 2007), neurons that are selectively activated when rodents moved into a specific location within a maze (O’Keefe and Dostrovsky, 1971). A closer analysis has shown that the DG place cells present multiple place fields (Jung and McNaughton, 1993; Leutgeb et al., 2007) and, like CA1 place cells, can remap (Leutgeb et al., 2007). Remapping is a property consisting of the change of the place cell firing pattern in response to a small change in the sensory (Muller and Kubie, 1987; Colgin et al., 2008) or behavioral context (Colgin et al., 2008). In this way, it has been suggested that this remapping property allows the encoding of information emerging from similar experiences into distinct neuronal representations, which in turn is important for pattern separation. However, one of the disadvantages of the data obtained with electrophysiological recordings is the difficulty in distinguishing between other neurons that are also glutamatergic-like mossy cells (MCs; Soriano and Frotscherf, 1994). In this scenario, neurophotonic techniques have allowed researchers to separate the contribution of GCs and MCs to pattern separation.

The DG is composed primarily of GCs, whose dendrites are arranged within the molecular layer, and their cell bodies form the adjacent GC layer (Amaral et al., 2007). Between the molecular layer and the CA3 region, there is a polymorphic layer called the hilus. Mossy cells are located only in the hilus region (Figure 1). However, because GCs fibers and MCs coexist in this region (Scharfman, 2016), their differential contribution to the circuitry functionality has been difficult to be dissected. One possible, and elegant, setting used for the study of the differential contribution from GCs and MCs has been to perform simultaneous optical stimulation and electrophysiological recordings (Senzai and Buzsáki, 2017; Jung et al., 2019). In these studies, an optrode was used. This array allows the simultaneous recording of the voltage field while the light is delivered to the tissue, making possible to see the instantaneous effect of light in the firing rate of units recorded (Royer et al., 2010; Anikeeva et al., 2011). Senzai and Buzsáki (2017) used dopamine receptor D2 (DRD2) promoter to drive the expression of a chloride pump called archaerhodopsin specifically to MCs. Using this strategy, neurons that suppressed their activity during the optical stimulation were classified as putative MCs. Surprisingly, this study found that MCs from the DG present one or more than one place fields, like GCs, making it necessary to reinterpret previous works (Jung and McNaughton, 1993; Leutgeb et al., 2007), but also proving the strength of the new optical techniques.

With this system, the accuracy of the study of the MCs’ electrophysiological characteristics can be enhanced. However, this form of identification presents some limitations that depend on neuronal connectivity. For example, in some cases, neurons that express archaerhodopsin can activate other neurons. Then, optical stimulation can induce suppression of the activity of neurons that express archaerhodopsin but excitation of neurons that are subsequently activated by them (Senzai and Buzsáki, 2017; Morales et al., 2019). This method does not allow the identification of GCs (that excite directly the MCs) but is useful for the identification of MCs. To tackle this issue, Danielson et al. (2017) used *in vivo* two-photon calcium imaging in awake behaving mice to differentiate the role of MCs and GCs. To achieve selective manipulation of MCs, they took advantage of the anatomical properties of MCs. Specifically, they injected an AAV-expressing Cre-recombinase into the DG. Then they injected a Cre-dependent rAAV-expressing GCaMP6f, a sensitive fluorescent protein used for imaging of neuronal activity, in one of the DGs. Because MCs project to contralateral DG, this technique allows the expression of fluorescent markers only in MCs of the contralateral DG. Then a chronic imaging window was implanted above the DG to visualize Ca^2+^ activity from MCs in head-fixed mice that have to run on a treadmill in different linear environments to receive a reward. They found that MCs have place fields, as has been previously described, but while GCs have high tuning specificity, MCs have low tuning specificity indicating that MCs have multiple firing fields. This supports the finding that place cells with multiple place fields that were found in electrophysiological experiments (Jung and McNaughton, 1993; Leutgeb et al., 2007) are principally MCs. Danielson et al. (2017) showed that the fraction of place coding was bigger in MCs than in GCs, supporting the idea that, in electrophysiological recording, MCs represent an important number of place cells. Although a powerful approach, a disadvantage of this setting is that the cell specificity is given by anatomical properties. Some studies show that MCs are not the unique cells that project to contralateral DG (Scharfman, 2018). If this is confirmed, the results reviewed above required to be interpreted with caution.

The results of a recent study (Jung et al., 2019) suggest a possible driver role of MCs in remapping. They showed that when there are changes in the environment, such as those used to induce remapping, MCs’ response precedes the activity change on GCs. It is important to note that, to differentiate between GCs and MCs, Jung et al. (2019) injected a CRE-inducible AAV to drive the expression of Chronos, an excitatory opsin that is faster and more light-sensitive than the conventional channelrhodopsin (Klapoetke et al., 2014), in two different transgenic mice line, DRD2-Cre and POMC-Cre mice, which allowed them to excite, MCs and GCs, respectively. Although the use of this setting to differentiate the role of MCs and GCs in the DG circuit was previously used, they argued that excitation is better than inhibition to discriminate between neuronal types.

In summary, the evidence described above suggests that both CGs and MCs may be deferentially involved in the functionality of the DG circuitry. In particular, MCs showed more sensibility to contextual changes than GCs suggesting that MCs could be part of the circuits that detect and encode the nonoverlapped information, while the GCs could be encoding the overlapped information. Although further research would be needed to completely dissect their specific function, the evidence accumulated until now proposes to MCs as important players in the mechanism of pattern separation.

### What Can We Say About the “Irritable” Hypothesis Using Neurophotonic Techniques?

One of the most exciting topics on the study of the DG is related to the role of each cell type in terms of which node orchestrates the activity of the other nodes. It has been shown that MCs can excite GCs directly and can inhibit GCs indirectly through the recruitment of feedback inhibition (Scharfman, 1995; Larimer and Strowbridge, 2008). However, because of the complexity of the DG circuit, the neuronal mechanisms underlying the net effect on GC activity are still a controversial matter (Scharfman, 2018).

In the early 1980s, some seminal studies showed that the stimulation of commissural (fiber of MCs) just before stimulating the perforant path (PP) produced an inhibition over the GC spikes population (Buzsàki and Eidelberg, 1981; Douglas et al., 1983), suggesting that the activation of MCs principally inhibits the activity of GCs. Interestingly, this conclusion was confirmed by subsequent experiments (Sloviter, 1983, 1991; Scharfman, 1995), allowing the establishment of the “dormant basket cells (BCs)” hypothesis. This hypothesis proposed that the net effect of MCs on GC activity was mediated by the activation of parvalbumin (PV+) GABAergic interneurons within the DG that then inhibit GCs (Sloviter, 1991). However, in contrast to this theory, other experiments suggested that the net effect was mediated by the excitation of the GCs by the MCs (Buckmaster et al., 1996; Ratzliff et al., 2004). This alternative hypothesis, called the “irritable mossy cells” hypothesis, proposed that MCs’ hyperexcitability increases the activity of the GCs affecting in this way the net effect onto the DG (Santhakumar et al., 2000). Despite that the last hypothesis was described in pathological conditions such as epilepsy, it has been extrapolated to memory function. Then, based on these two perspectives, MCs could modulate GCs’ response by indirect inhibition or direct activation.

One of the problems with electrical stimulation is how to selectively and specifically stimulate MCs (Amaral et al., 2007; Leranth and Hajszan, 2007). Then, identification of MCs by other parameters than their electrophysiological properties is required. Jackson and Scharfman (1996) used in hippocampal slice the voltage-sensitive dye technique (90–92). This technique has a better spatial resolution than traditional electrophysiological approaches and permits to study the spread of activity within DG after stimulation of PP. They showed that the spread of activity depends specifically on the hilar activation, whereas the PP damage was not related to this outcome (Jackson and Scharfman, 1996) and that electrical stimulation of the hilus induces depolarization at the inner molecular layer. Thus, they suggested that the spread of activity delivered by PP stimulation depends on positive feedback between GCs and MCs. Despite these results, other investigations that combine a laser-scanning photostimulation with a voltage-sensitive dye (Xu et al., 2010) have shown that photostimulation of the hilus does not increment the activity in GCs (Sun et al., 2017). Hsu et al. (2016) performed a series of experiments to resolve the discrepancy between these results. They injected unilaterally a CRE-inducible AAV carrying the ChR2 gene in the hilus of Grik4-cre hemizygous mice to direct the expression of ChR2 to the commissural fiber of contralateral DG. The authors found that inhibition/excitation balance in GCs was increased when commissural fibers were photostimulated. Moreover, they showed that while concurrent activation of commissural and perforant pathways increased the response of GCs, the delayed activation of PP compared with the commissural pathway decreased the percentage of responding GCs (Hsu et al., 2016). These results proposed that the optogenetic stimulation of the PP at 10 Hz, the “dormant BCs” hypothesis, seems to apply. Interestingly, the frequency of the optostimulation is quite similar to the one observed in the HP of animals during active exploration periods.

Contrasting with what was described above, another study (Hashimotodani et al., 2017) decided to address the effect of fast (30 Hz) optical stimulation of MC fiber, because this kind of stimulation can induce long-term potentiation (LTP). To generate an optical fast stimulation, they used ChIEF, a faster version of ChR2 (Lin et al., 2009). They found that fast optical stimulation induces LTP between MCs–GCs synapses, but not in mossy-interneurons synapses. This facilitation increases the excitation/inhibition balance, thereby inducing an increment in GC activity. These results obtained using a fast optostimulation protocol support the “irritable” hypothesis. Taking all this evidence together, the optostimulation frequency performed at the PP seems to be critical in defining the role of the MCs in the modulation of the GC activity. If the input is low frequency, the indirect inhibition mechanism seems to modulate the GC net effect, whereas the direct MCs–GCs excitation seems to be more preponderant when higher-frequency inputs impact into the circuit.

## Neurophotonics Applications in the Study of the DG Interneurons Role in Pattern Separation

Several models have proposed that GABAergic DG interneurons mediate the control of GC excitability and the orthogonalization of engrams that represents similar contexts (Rolls, 2013). Electrophysiological experiments have shown that GABAergic interneuron activity in the DG, unlike other hippocampal regions, is higher in a novel than in a familiar context (Nitz and McNaughton, 2004), suggesting its role in encoding novel information (Figure 1). Some of the GABAergic interneurons that contact GCs at the perisomatic region are BCs, whose inputs come from other GCs, the perforant pathway (Freund and Buzsáki, 1996), and MCs (Scharfman and Myers, 2012; Hsu et al., 2016; Scharfman, 2016; Danielson et al., 2017). Thus, in this way, these interneurons could control both feedback and feedforward inhibition onto GCs (Freund and Buzsáki, 1996; Savanthrapadian et al., 2014). On the other hand, within interneurons that contact the dendritic region of GCs are the hilar PP-associated interneurons (HIPPs), which correspond to a type of interneurons that have their soma in the hilus, where contact with GC axons takes place (Freund and Buzsáki, 1996; Savanthrapadian et al., 2014; Yuan et al., 2017). It has been proposed that HIPPs could control GC activity through feedback mechanisms (Houser, 2007). There has been an established relationship between the anatomical characteristics of these subpopulations and the presence of specific neuronal markers (Freund and Buzsáki, 1996; Savanthrapadian et al., 2014). The expression of neuronal markers associated with distinct GABAergic cells has allowed the use of optogenetics techniques to analyze the role of each of these GABAergic interneurons in DG networks and pattern separation. Specially, BC interneurons are PV+, whereas HIPP interneurons are SOM+ (Freund and Buzsáki, 1996; Savanthrapadian et al., 2014; Yuan et al., 2017).

The stronger inhibition mediated by the recruitment of PV+ interneurons counterbalances excitation of DG networks. In this way, stronger excited cells recruit GCs more effectively than less excited cells, allowing a “winner-takes-all” situation that would allow a good pattern separation mechanism (Sambandan et al., 2010; Guzman et al., 2019). Electrophysiological experiments have shown that this property depends on the coactivation of the perforant pathway and mossy fibers (Sambandan et al., 2010). Although there are other mechanisms capable of regulating the activity of PV+ interneurons, Hu et al. (2010), using confocal imaging and patch-clamp simultaneously, showed that some of the intrinsic properties of PV+ interneurons dendrites, such as the presence of Kv3 channels, are implicated in the rapid and precise time inhibition mediated by PV+. On the other hand, several studies (Savanthrapadian et al., 2014; Yuan et al., 2017) showed that SOM+ also contributes to the precision of the discharge of PV+. In this line of evidence, Savanthrapadian et al. (2014) injected a Cre-inducible rAAV vector containing ChR2-tdT into the DG of SOM-Cre mice. They studied the PV+ interneurons, while paired optical stimulation of SOM+ interneurons with electrical stimulation of PP. They showed that the optical stimulation of the outer molecular layer, where the axons of HIPPs are present, increases the precision of action potential generation in PV+ interneurons. Yuan et al. (2017) showed that there are two types of SOM+ interneurons within the DG, the HIPP interneurons, which were studied by Savanthrapadian et al. (2014), and SOM+ interneurons that have their axons in the hilus and contact other interneurons such as PV+ interneurons. This last group is called hilus-associated interneuron (HIL; Yuan et al., 2017). In this study, the scientists used a similar injection protocol as described by Savanthrapadian et al. (2014), but besides stimulating the outer molecular layers for the recruitment of HIPPs, they stimulated the perisomatic region of PV+ interneurons. Using this approach, they showed that the activity of the HIL determines the activity of PV+ interneurons (Yuan et al., 2017). Thus, the activity of PV+ interneurons is regulated by SOM+ interneurons through dendritic inhibition by HIPPs and perisomatic inhibition by HIL (Figure 1). This complex array of inhibitory control seems to indicate a complementary role between PV+ and SOM+ interneurons and could be instrumental for pattern separation.

The EC is generally characterized as the main input to the DG (Rolls, 2013). Understanding how cortical inputs modulate DG inhibitory microcircuits is crucial to understand the processing of information in the HP. To this end, Lee et al. (2016) studied how PV+ and SOM+ interneurons affect the activity of GCs in response to cortical stimulation. They injected an AAV (AAV5)-expressing Cre-dependent enhanced halorhodopsin (eNpHR3.0). Using this experimental approach, they selectively inhibit each of these interneurons, PV+ and SOM+. They showed that inhibition of PV+ interneurons suppresses GCs’ responses to single cortical stimulation. When cortical stimulation was in theta (θ) or gamma (γ) frequencies (Lee et al., 2016), that is, frequencies present during exploration (Bragin et al., 1995), they found that both types of interneurons differentially regulate GCs’ responses. Interestingly, they found that PV+ regulates the onset of the spike series, whereas SOM+ interneurons regulate principally late spikes. Overall, these results are in agreement with the view that PV+ and SOM+ interneurons play complementary roles in pattern separation.

Besides regulating GC excitability, it is possible that GABAergic interneurons also participate in the orthogonalization of engrams that represents similar contexts through a lateral inhibition mechanism. By coupling the expression of td-tomato or enhanced green fluorescent protein (EGFP) reports with the expression of neurochemical marker for its identification in slice experiments, Espinoza et al. (2018) found that, in the case of GCs-PV+ connection, the ratio of lateral inhibition regarding recurrent inhibition was higher, suggesting an important role of this interneurons in lateral inhibition. On the other hand, Stefanelli et al. (2016) were interested in the size of the ensemble recruited during the encoding of contextual information and how it modulates the specificity during recall. To tackle this question, they expressed ChR2 in GCs, SOM+, and PV+ interneurons to optostimulate these cells during the encoding of a contextual fear memory paradigm. They showed that the rise time of GABAergic current response induced by PV+ stimulation was the shortest, whereas the rise time of GABAergic current response induced by SOM+ and GCs did not have significant differences. In this way, the authors conclude that, because of the similarity between GABAergic current response induced by SOM+ and GCs, the lateral inhibition induced by GCs corresponds to the recruitment of SOM+ interneurons (Stefanelli et al., 2016). Thus, in the case of orthogonalization, experimental evidence suggests that PV+ and SOM+ participate in a complementary way. These results provide evidence that integrates the role of different DG cell types in the memory allocation and how it could contribute to the pattern separation process. From this perspective, DG interneurons are recruited during the encoding of contextual fear memory. Their role during this process seems to be circumscribed to the control of the size of the ensemble. If this process is affected by blocking the activity PV+ or SOM+ cells, the number of recruited GCs would increase. This outcome could affect the selectivity of the storage and/or recall of the information because the probability of overlap with other neuronal ensembles coding other memories is enhanced.

## Neurophotonics Techniques to Understand the Role of Adult-Born Granule Cells in Pattern Separation

The DG circuit, as well as the olfactory bulb, is continuously changing because of the integration of adult-born GCs (abGCs; Sahay et al., 2011b). A growing body of studies are currently focused on finding if abGCs play a particular role in pattern separation. Clelland et al. (2009) found that blocking hippocampal adult neurogenesis by X-ray irradiation altered the animal’s ability to distinguish small changes in spatial discrimination, but not unmistakable changes. Consistently, Sahay et al. (2011a) observed that animals with genetically increased levels of adult neurogenesis were better at discriminating between two similar contexts (Freund and Buzsáki, 1996; Sahay et al., 2011a). Moreover, many studies suggested that these new neurons could be a preferential substrate for remapping the place cells in presence of subtle changes in the environment. This is because the immature granular neurons have higher excitability and plasticity that distinguishes them from the population of old and relatively silent neurons (Espósito et al., 2005; Marín-Burgin et al., 2012). In addition to this, it has been proposed that mature neurons could be specialized for certain, more stable characteristics of their environment because they would respond preferentially to the inputs they received during their development (Aimone et al., 2011). On the other hand, immature GCs showed a low threshold for the induction of LTP (Schmidt-Hieber et al., 2004; Ge et al., 2007). Then, the particular properties of immature GCs confer them the characteristics required to be involved in pattern separation. Consistently with this idea, Nakashiba et al. (2012) suggested that neither the larger number nor the more dispersed activity of the DG is sufficient to separate similar contexts and that young aGCs would be necessary to allow this process.

Ikrar et al. (2013) studied the DG response to electrical stimulation with a voltage-sensitive dye technique (Ebner and Chen, 1995; Chemla and Chavane, 2009; Tsytsarev et al., 2014) using an iBax-nestin mice, a model mice in which neurogenesis can be enhanced with tamoxifen administration. They showed that photostimulation or electrical stimulation of DG induced a smaller and less-spread neuronal excitability in mice with increased adult neurogenesis compared to the controls (Ikrar et al., 2013). These results suggest that adult neurogenesis is an important factor in the control of the DG neurons’ excitability. This result was supported by a different studies (Temprana et al., 2015; Drew et al., 2016). In this case, a retrovirus expressing a light-activated channel ChR2 was delivered to the DG of adult mice for its selective transduction in neural progenitor cells of the adult DG. Then, acute slices were prepared some weeks postinjection for studying the effect of photostimulation of abGCs generated at different time points. They showed that abGCs activate hilar GABAergic interneurons that in turn inhibit mature GCs (Figure 1). Specifically, Temprana et al. (2015) showed that recruitment of feedback inhibition is higher in abGCs of 7 weeks than in abGCs of 4 weeks. This result suggests that as time passes abGCs tend to be more integrated into inhibitory circuits that facilitate their role in controlling the excitability of surrounding neurons.

A recent study (Luna et al., 2019) showed that, besides the recruitment of feedback inhibition by abGCs (Temprana et al., 2015; Drew et al., 2016), these newborn neurons can directly inhibit mature GCs. Specifically, Luna et al. (2019) selectively expressed archaerhodopsin T in abGCs. They showed that optical inhibition of abGCs produced an increment in the DG LTP response to electrical stimulation, even when GABA antagonists were used. This could indicate that inhibition is independent of GABAergic interneuron activation in the hilus. They also studied the effect of abGC activation in mature GCs, by selectively expressing ChR2 in abGCs. They showed that low intensities of light, which produce low levels of glutamate release from abGCs, induce IPSPs in mature GCs. Moreover, high intensities of light, which produce high levels of glutamate liberation, induce EPSP in mature GCs. Finally, they showed that low glutamate liberation, that is, IPSP in mature GCs, is due to the preferential activation of the lateral EC that carries contextual information (Hargreaves et al., 2005; Wilson et al., 2013). Keeping low levels of mature GC excitability is important for pattern separation (Jinde et al., 2012; Sahay et al., 2011a). Luna et al. (2019) suggested that contextual information—besides spatial information—is relevant to promote a sparse coding in DG. Consistently, using calcium imaging, Danielson et al. (2016) differentiate the activity of abGCs from other populations that present a low spatial tuning but are good novelty detectors, supporting a fundamental role of abGCs in disambiguating contextual information through the process of pattern separation.

All the evidence described in this section proposed that abGCs could play a key role in the formation of orthogonal representations from similar inputs. This is because their high excitability during the early stages of their development is critical to determine which inputs will recruit them subsequently. Because neurogenesis is a continuous process, there are always abGCs at different stages of development. Therefore, the probability that two different experiences recruit the same subset of abGCs at the same developing time is low. This characteristic gives them a potential role in orchestrating the rest of the cells that potentially form the differentiated engrams in the DG.

## Final Remarks

Neurophotonic techniques allowed the study of the role of different neuronal types in the DG networks functionality. Specifically, the evidence described above suggests a differential role of each neuronal type in the mechanisms underlying pattern separation. As we have described, neurophotonic studies led to propose models that go beyond unique neuronal types for information processing and where several elements of DG network share complementary roles in the differentiation of overlapping information. Based on the body of evidence presented above, we are proposing a possible way in which all these different cell types might interact and contribute to pattern separation.

Neurophotonics have contributed to differentiate the role of three types of DG glutamatergic cells, MCs, newborn GCs (abGCs), and mature GCs. Thus, it has been shown that MCs and abGCs present more remapping than mature GCs (Danielson et al., 2016, 2017; Senzai and Buzsáki, 2017), which suggests that MCs and abGCs are more sensible than GCs to detect small environmental changes, that is, when differences must be detected in similar episodes. Besides, neurophotonics experiments suggest that these neuronal types would respond before than GCs (Marín-Burgin et al., 2012; Jung et al., 2019), suggesting that both neuronal types could initiate the process of pattern separation. Besides, both neuronal types are more sensitive to contextual changes (Danielson et al., 2016, 2017; Senzai and Buzsáki, 2017; Luna et al., 2019), which means that contextual information would be more relevant than other type of information. Thus, MCs and abGCs could initiate pattern separation through the detection of environmental changes, especially changes in contextual information. After activation of MCs and abGCs, they can initiate an inhibitory network. While abGCs can inhibit directly GCs (Luna et al., 2019), MCs and abGCs would activate PV (Scharfman, 2018; Groisman et al., 2020), which in turn produces a lateral inhibition proposed to be important for pattern separation (Espinoza et al., 2018). Interestingly, the activity of SOM modulates the activity of PV (Savanthrapadian et al., 2014; Yuan et al., 2017) and can produce itself a lateral inhibition (Stefanelli et al., 2016). On the other hand, the evidence indicates that the activity of SOM is delayed when compared with the activity of PV (Hsu et al., 2016; Stefanelli et al., 2016). Thus, both PV and SOM interneurons control the activity of GCs, but likely in a different time with PV activity preceding SOM activity. Some models propose pattern separation mechanisms that take into account and emphasize the role of PV interneurons (Guzman et al., 2019), abGCs (Sahay et al., 2011b), and MCs (Nakazawa, 2017). In this work, we make a complementary interpretation to all these models, paying special attention to the interaction between the different cell types present in the DG (Figure 1). Still, more work is required to better understand the mechanism, dynamics, and constraints of pattern separation in the DG.

However, it is important to highlight that in the last years our understanding of this process advanced enormously, thanks to the development of neurophotonic techniques. We believe that the continuous advancement in this field in combination with genetic tools will prove to be a powerful strategy for ll for modeling pattern separation where a complementary role of different types could be studied.

## Author Contributions

CM, JM, MM, FG, PB, and NW conceived the content, wrote and organized the manuscript.

## Conflict of Interest

The authors declare that the research was conducted in the absence of any commercial or financial relationships that could be construed as a potential conflict of interest.
